# Dissemination of novel Tn*7* family transposons carrying genes for synthesis and uptake of fimsbactin siderophores among *Acinetobacter baumannii* isolates

**DOI:** 10.1099/mgen.0.000548

**Published:** 2021-03-22

**Authors:** Mohammad Hamidian, Ruth M. Hall

**Affiliations:** ^1^​ iThree Institute, University of Technology Sydney, Ultimo, NSW 2007, Australia; ^2^​ School of Life and Environmental Sciences, University of Sydney, NSW 2006, Australia

**Keywords:** *Acinetobacter baumannii*, global clones, homologous recombination, iron acquisition, siderophore, Tn*7 *family transposon

## Abstract

*
Acinetobacter baumannii* is a successful opportunistic pathogen that can compete for iron under iron-limiting conditions. Here, large novel transposons that carry genes for synthesis and transport of the fimsbactin siderophores present in some *A. baumannii* strains were examined. Tn*6171*, originally found in the *A. baumannii* global clone 1 (GC1) lineage 2 isolate D36, includes *tns* genes encoding proteins related to the TnsA, TnsB, TnsC transposition proteins (50–59 % identity), TnsD targeting protein (43 % identity) and TnsE (31 % identity) of Tn*7*, and is found in the chromosome downstream of the *glmS* gene, the preferred location for Tn*7*, flanked by a 5 bp target site duplication. Tn*6171* is bounded by 29 bp inverted repeats and, like Tn*7*, includes additional TnsB binding sites at each end. Tn*6171* or minor variants were detected in the equivalent location in complete or draft genomes of several further *A. baumannii* isolates belonging to GC1 [sequence type (ST) 1, ST81, ST94, ST328, ST623, ST717], GC2 (ST2) and ST10. However, in some of these isolates the surrounding *glmS* region was clearly derived from a different *A. baumannii* lineage, indicating that the transposon may have been acquired by replacement of a segment of the chromosome. A recombination-free phylogeny revealed that there were several transposon acquisition events in GC1. The GC1 isolates were mainly lineage 2, but a potential third lineage was also detected. A related transposon, designated Tn*6552*, was detected in ATCC 17978 (ST437) and other ST437 isolates. However, the Tn*6552 tnsD* targeting gene was interrupted by an ISAba12, and Tn*6552* is not downstream of *glmS*.

## Data Summary

Complete and draft *Acinetobacter baumannii* genomes (3575 entries) were retrieved from the National Center for Biotechnology Information (NCBI) GenBank nonredundant and Whole Genome Shotgun (WGS) databases screened and analysed here. The full strain list and the ftp addresses used to retrieve the genomes are publicly available at https://www.ncbi.nlm.nih.gov/genome/?term=Acinetobacter+baumannii. Individual genome accession numbers used in this study are included either in the text, Table 1 or Table 2.

**Table 1.** Properties of strains carrying a Tn*6171* transposon or variant Complete genomes are highlighted in grey.

Strain	Year	Country	Source	ST	GC	Tn*6171* variant	Size (bp)	TSD	*glmS* allele	GenBank acc. no.
D36	2008	Australia	Wound	81	1	Tn*6171*	49 915	AAGGC	1	CP012952
6013150	2007	UK	Skin	81	1	Tn*6171*	49 915	AAGGC	1	ACYQ
6013113	2007	UK	Skin	81	1	Tn*6171*	49 915	AAGGC	1	ACYR
MRSN 3527	2011	USA	Wound	81	1	Tn*6171*	49 915	AAGGC	1	JPHZ
PR332	nr	USA	nr	81	1	Tn*6171*	49 915	AAGGC	1	NGDV
ABS029	2012	Iran	Wound	328	1	Tn*6171*	49 915	AAGGC	1	WIOH
ABS062	2012	Iran	Wound	328	1	Tn*6171*	49 915	AAGGC	1	WIOF
ABS063	2012	Iran	Wound	328	1	Tn*6171*	49 915	AAGGC	1	WIOE
ABS122	2013	Iran	Wound	328	1	Tn*6171*	49 915	AAGGC	1	WIOA
ABS201	2013	Iran	Wound	328*	1	Tn*6171*	49 915	AAGGC	1	VJZY
R1B	2011	Saudi Arabia	nr	1	1	Tn*6171*	49 915†	AAGGC	1	JICK
AB4052	2016	USA	Wound	2	2	Tn*6171*	49 915†	AAGGC	623	LRED
Pesh-31	2015	Pakistan	Pus	2	2	Tn*6171*	49 915†	AAGGC	623	QQQA
ABBL062	2008	USA	Blood	2	2	Tn*6171*	49 915†	AAGGC	623	LLFH
ABBL040	2007	USA	Blood	2	2	Tn*6171*	49 915†	AAGGC	623	LLDU
MRSN6273	2012	USA	Sputum	94‡	1	Tn*6171*§	49 915†	AAGGC	623	LNCY
MRSN6269	2012	USA	Sputum	94	1	Tn*6171*§	49 915†	AAGGC	623	LNCX
MRSN5540	2011	USA	Blood	94	1	Tn*6171*§	49 915†	AAGGC	623	LNCW
MRSN3941	2011	USA	Wound	94	1	Tn*6171*§	49 915†	AAGGC	623	LNCV
MRSN4106	2011	USA	Wound	94	1	Tn*6171*§	49 915†	AAGGC	623	JPHX
MRSN3942	2011	USA	Wound	94	1	Tn*6171*§	49 915†	AAGGC	623	JPHY
MRSN3405	2011	USA	Wound	94	1	Tn*6171*§	49 915†	AAGGC	623	JNOU
MRSN4119	2011	USA	Wound	94	1	Tn*6171*§	49 915†	AAGGC	623	LNBZ
600	2011	UK	nr	94	1	Tn*6171*§	49 915†	AAGGC	623	CBXD
ARLG1323	<2017	nr	nr	10	–	Tn*6171*§	49 915†	AAGGC	10	NGHP
AbPK1	2012	Pakistan	Sheep lung||	2	2	Tn*6171*::IS*Aba*1¶	51 200	AAGGC	2	CP024576
5457	<2019	India	nr	623**	1	Tn*6171-v1*††	49 124	AAGGC	623	CP045541
ZQ3	2016	Iraq	Blood	717	1	Tn*6171-v1*	49 124†	AAGGC	1	PHJZ
G21	2011	Australia	nr	2	2	Tn*6171-v1*	49 124†	AAGGC	623	UCOP
AB309	2013	Saudi Arabia	Blood	2	2	Tn*6171-v1*	49 124†	ACTAG	2	LXTY
AB552	2013	Saudi Arabia	Blood	2	2	Tn*6171-v1*	49 124†	ACTAG	2	LXUE
VB2486	2019	India	Sputum	1	1	Tn*6171-v2*	52 602	ACAGC	437	CP050403
VB24319	2017	India	Blood	1	1	Tn*6171-v2*	52 602†	ACAGC	437	RHLV
VB29123	2017	India	Blood	1	1	Tn*6171-v2*	52 602†	ACAGC	437	RBVT

*ST328 is a single locus variant of ST81 within GC1.

†Estimated.

‡ST94 is a double locus variant of ST1.

§Includes an extra 8 bp repeat unit in the segment between IS*1236-var* and IS*Aba*12.

||Sheep bronchoalveolar lavage.

¶Contains an IS*Aba*1 copy and a repeat sequence of 120 bp, in the middle of the segment between IS*Aba*12 and ∆IS*123*-*var*, consisting of 15×8 bp units. D36 includes only three units (of 8 bp).

**ST623 is a single locus variant of ST1 representing the majority of GC1 strains.

††Tn*6171*-v1 includes a 610 bp IS*Aba*12-mediated deletion.

nr, Not recorded.

**Table 2.** Properties of ST437 strains that carry Tn*6552*

Strain	Year	Country	Source	Size (bp)	In *glmS/*TSD	GenBank acc. no.
ATCC 17978	1951	nr	Meningitis	45 140	N/GCTTC	CP012004
XH181	2014	China	nr	45 140*	N/GCTTC	MDWH
XH182	2014	China	nr	45 140*	N/GCTTC	MDWJ
XH183	2014	China	nr	45 140*	N/GCTTC	MDWK
XH184	2014	China	nr	45 140*	N/GCTTC	MDWL
XH191	2014	China	nr	45 140*	N/GCTTC	MDWG
XH192	2014	China	nr	45 140*	N/GCTTC	MDWI
XH193	2014	China	nr	45 140*	N/GCTTC	MDWF
XH198	2014	China	nr	45 140*	N/GCTTC	MDWM

nr, Not recorded.

*Estimated.

**Impact Statement**

*
Acinetobacter baumannii* strains have emerged as successful opportunistic pathogens that have become difficult to treat because of acquisition of resistance to most or all of the available antibiotics suitable for treatment. Their ability to resist extreme environmental conditions and to compete for micronutrients required for growth, such as iron, contributes to their success. The transposons reported here could increase the iron-uptake capacity of isolates that carry them by supplying the genes required for synthesis and uptake of the fimsbactin group of siderophores. As Tn*7* relatives, they target the region downstream of the chromosomal *glmS* gene, allowing them to spread without deleterious effects. However, replacement of the chromosomal *glmS* region with an equivalent segment from a different strain carrying the transposon also has contributed to this spread. One transposon has been acquired by different routes in more than one lineage of the dominant global clone 1 (GC1) and GC2 clonal complexes.

## Introduction

The Gram-negative opportunistic pathogen *Acinetobacter baumannii* is mainly associated with nosocomial infections, including pneumonia, blood stream, urinary tract and wound infections [1]. Infections caused by two globally distributed clones of *A. baumannii *, namely global clone 1 and 2 (GC1 and GC2), have been responsible for the majority of treatment difficulties due to high levels of resistance to multiple antibiotics [1–4]. However, beyond drug resistance, various virulence mechanisms, such as biofilm formation or production of surface polysaccharides that protect this organism from the immune system or enable it to compete with its host for micronutrients, have been identified [5, 6]. In addition, the success of *A. baumannii* is partly attributable to its ability to survive, persist and thrive in the health-care environment [5].

Amongst the virulence factors identified to date, siderophore-mediated iron-acquisition systems are known to be of major importance [7–9]. Iron is an important micronutrient for all bacterial pathogens. In the human or animal host, free iron is generally found bound to molecules such as transferrin and haem and, therefore, not readily available. Bacterial pathogens secrete ferric-binding compounds, called siderophores, that allow them to take up and utilize iron. Iron-uptake systems are encoded and expressed by most *A. baumannii* clinical isolates [7, 9–11], giving the organism the ability to grow under iron-limiting conditions [7–9]. To date, three siderophore systems for iron acquisition have been identified in *A. baumannii* with gene clusters for two of them, acinetobactins and baumannoferrins [12], found in most *A. baumannii* genomes examined so far [6, 8–12]. However, a third gene cluster, now known to be responsible for the synthesis and transport of fimsbactins [13], was found only in ATCC 17978 [8, 9, 13, 14]. This cluster includes 15 genes involved in siderophore biosynthesis, 3 genes involved in recognition and uptake of the ferric siderophores, and 2 genes encoding putative efflux pumps, a putative MFS efflux pump and the second efflux pump, which is a member of the multidrug and toxic compound extrusion (MATE) family [8, 9, 13].

We previously reported the complete genome sequence (GenBank accession no. CP012952) of an extensively antibiotic-resistant isolate, D36 [15], that belongs to lineage 2 of the GC1 clonal complex [4]. It was noted that the chromosome of D36 carries a 49.9 kbp transposon, designated Tn*6171*, which carries a siderophore synthesis gene cluster previously seen only in ATCC 17978 and also encodes transposition proteins related to those of Tn*7* [15].

Here, we examine the properties of transposon Tn*6171* and related transposons that carry genes for production of fimsbactin siderophores and are related to Tn*7*, one of the most well-studied bacterial transposons [16–18]. The distribution and location of these transposons in publicly available complete genomes of *Acinetobacter* strains in the National Center for Biotechnology Information (NCBI) GenBank non-redundant database and in draft genomes in the Whole Genome Shotgun (WGS) database were also examined. To assess whether they were acquired by transposition or were imported *in situ*, the surrounding chromosomal region was compared to that of standard examples of the appropriate clonal lineage.

## Methods

### Sequences used in this study

Tn6*171*, found previously in the chromosome of the *A. baumannii* strain D36 (GenBank accession no. CP012952) [15], was used to study the transposon structure and distribution. Draft genome sequences of isolates recently assigned to lineage 2 of GC1 (which includes D36) [4, 19] were also searched for the presence of Tn*6171*. To analyse the structure of the transposon that encodes a siderophore cluster in *A. baumannii* ATCC 17978, a more recent complete genome sequence of this strain (available under GenBank accession no. CP012004) was used, given that the original genome sequence (GenBank accession no. CP000521) contains several sequencing and assembly errors [20]. The GenBank non-redundant database was searched to find additional siderophore-containing Tn*7* family transposons using low stringency blast and the sequence of the boundaries between Tn*6171* and the chromosome as the query. These genomes (GenBank accession numbers CP045541, CP024576 and CP012004) were retrieved from GenBank and studied here.

### Sequence annotation

Protein coding and gene features of transposons were annotated manually using a combination of blastp (http://blast.ncbi.nlm.nih.gov/Blast.cgi), Pfam (http://pfam.xfam.org/) and UniProt (https://www.uniprot.org) searches, as described elsewhere [21–23]. The fimsbactin genes were annotated in accordance with the names assigned recently [13]. The IS-Finder (https://www-is.biotoul.fr/) database was used to identify insertion sequences.

### Bioinformatics analysis

A range of bioinformatic tools were used for sequence analyses. A local database that consisted of 3575 draft genomes of *A. baumannii* that were publicly available, as of April 2019, in the WGS database and downloaded for a previous study [24], was also searched to find transposons that carry iron-uptake and siderophore systems. Sequence analysis was done locally using a stand-alone blast program available at https://ftp.ncbi.nlm.nih.gov/blast/executables/LATEST/. For genomes containing the Tn6*171* and variants, the sequence types (STs) [Institut Pasteur MLST (multilocus sequence typing) scheme] were determined using the *A. baumannii* MLST database website (https://pubmlst.org/abaumannii/). The chromosome of isolate A1 (CP010781), an early GC1 isolate [4, 25], was used for comparison of sequences found in GC1 isolates, and the chromosome of A320 (CP032055), an early GC2 isolate [26], was used for the GC2 genomes. The LAC-4 genome [27] was used as an ST10 standard. Artemis Comparison Tool (ACT) 16.0.0 [28] was used to visualize comparisons of large regions performed by stand-alone blast. Snap Gene Viewer v 4.2.4 was used to visualize, manipulate and export the sequence data. Figures were drawn to scale using SnapGene Viewer v 4.2.4 and reconstructed using the Inkscape v.1.0 program.

A recombination-free phylogenetic tree for GC1 was reconstructed as described previously [4, 19]. Genomes of all lineage 2 isolates and representative lineage 1 isolates were included. Relevant information on these isolates can be found in published references [4, 19]. The revised genome for AB307-0294 [20], found under GenBank accession number CP001172.2, was also included.

## Results

### Tn*6171*, a Tn*7*-family transposon that targets *glmS*

**Fig. 1.**
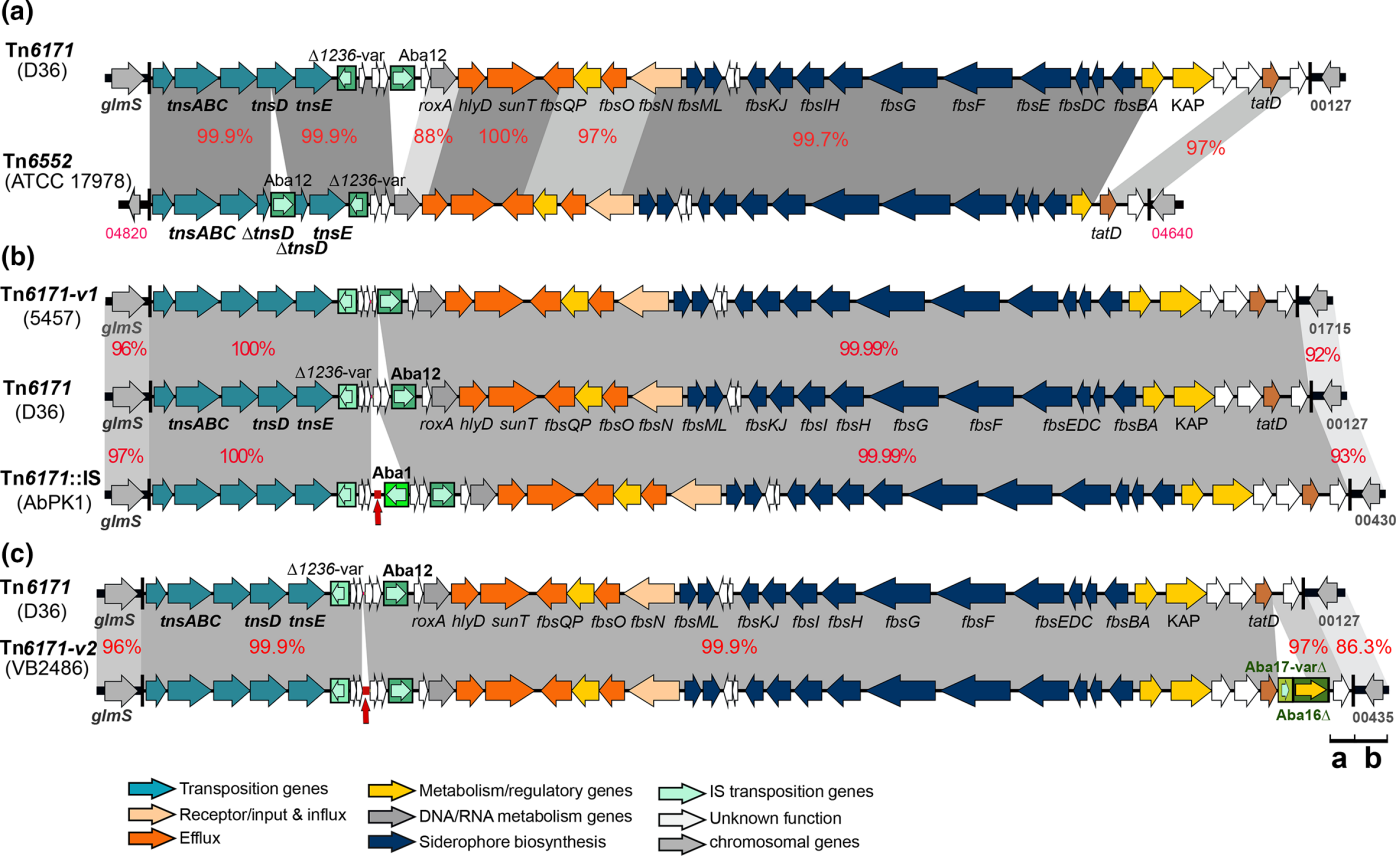
Genetic structure of transposons. Tn*6171* compared to (**a**) Tn*6552*, (**b**) Tn*6171-v1* and Tn*6171*::ISAba1, and (**c**) Tn*6171-v2*. Arrows show the extent and orientation of genes and ORFs with colouring indicating function (see the key). Insertion sequences are represented as green boxes with an internal arrow for their transposition gene(s). Red vertical arrows show the location of repeated 8 bp sequence. Shared segments with sufficient homology are shown using different shades of grey, with DNA per cent identities shown using red numbers. Drawn to scale form GenBank accession numbers CP012952 (Tn*6171*), CP012004 (Tn*6552*), CP032055 (Tn*6171*-v1), CP024576 (Tn*6171*::ISAba1) and CP050403 (Tn*6171*-v2).

**Fig. 2.**
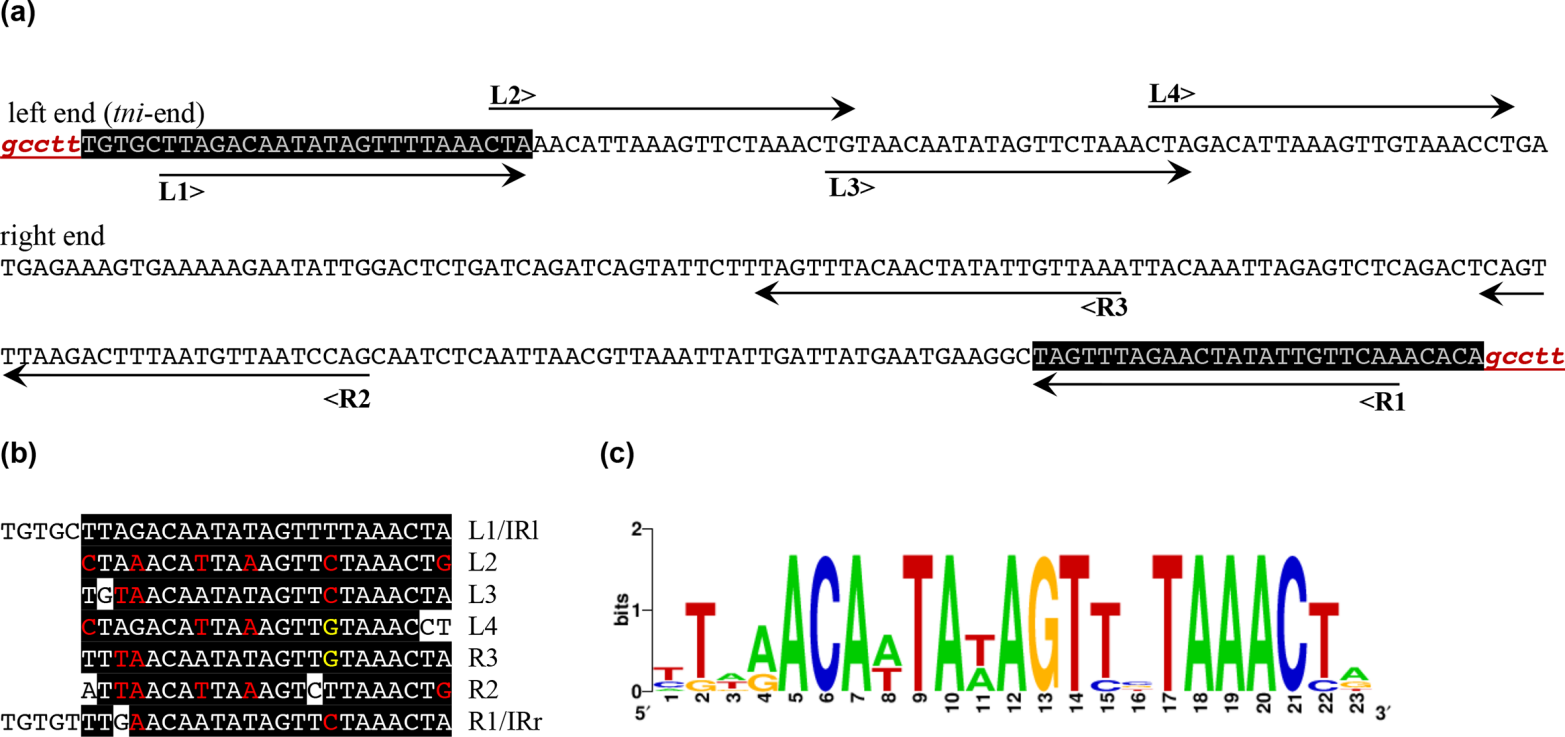
IR and transposon binding sites of Tn*6171*. (**a**) Sequence at the left and right end of Tn*6171* is shown in upper-case letters, and the flanking 5 bp TSD is in red lower-case letters. The extent of transposon binding sites is indicated using horizontal arrows numbered L1–4 and R1–3. (**b**) Alignment of the transposase binding sites. Conserved residues are shown using white letters on black, and partially conserved residues are indicated using red and yellow letters on black. (**c**) Sequence conservation of transposon binding sites presented by a WebLogo drawn online using software available at https://weblogo.berkeley.edu/logo.cgi.

The 49 915 bp Tn*7* family transposon in the GC1 strain D36, which was noted previously and named Tn*6171* [15], is located at bases 133 828 and 183 749 in the GenBank accession number CP012952 sequence (Fig. S1). Tn*6171* contains 36 genes ranging in size from ~600 bp to 2.1 kbp (locus_ids AN415_00128–AN415_00167), and four proteins encoded by genes at the left end (as shown in Fig. 1) share 50, 56, 59, 43 and 31% identity with the Tn*7* transposition proteins TnsA (required for second strand cleavage), TnsB (the transposase), TnsC (transposase accessory protein), TnsD (required for high frequency transposition to the preferred site downstream of *glmS*) and TnsE (required for broader dissemination), respectively. Here, Tn*6171* was found to be located 25 bp downstream of the *glmS* gene (locus_id AN415_00168 in CP012952) and flanked by a 5 bp target site duplication (TSD) AAGGC (bases 133 823–133 827 and 183 750–183 754 in CP012952). As this is the location targeted by Tn*7* [16, 17], this suggests that Tn*6171* uses the same targeting system as Tn*7*. Tn*6171* starts with 5′-TGT-3′ and ends with 5′-ACA-3′, and is bounded by 28 bp imperfect inverted repeats (IRs). In addition, several copies of the inner part of the IRs, equivalent to the binding sites for the transposase TnsB of Tn*7* [29], were found within 200 bp of the transposon boundaries. The configuration of these sites, four overlapping copies on the left (*tns* end) and three separated copies on the right, is the same as in Tn*7* (Fig. 2). All these properties are reminiscent of those of Tn*7* and confirm the assignment of Tn*6171* to the Tn*7* family.

Tn*6171* includes a copy of the insertion sequence ISAba12 and a 719 bp segment, which appears to be a remnant of a novel insertion sequence related to IS*1236* (Fig. 1a). In addition to the transposition genes, it contains genes encoding proteins with several predicted functions, including iron acquisition, metabolism, efflux pumps and regulatory (Fig. 1a, Table S1, available with the online version of this article). A large set of genes encode proteins related to ones produced by *Acinetobacter baylyi* ADP1 that have been shown to direct production of a series of related catechol/hydroxamate siderophores, which were named fimsbactins [13]. Hence, the *fbs* gene nomenclature used in that study [13] was adopted to annotate the siderophore genes in Tn*6171* (Table S1, Fig. 1).

### Tn*6171* is characteristic of the ST81 clade of GC2 lineage 2

We first examined whether Tn*6171* was also present in the genomes of other isolates that, along with D36, belong to the clade of GC1 lineage 2, which includes ST81 and ST328 isolates [4, 19]. All ST81 strains examined previously [4, 19], regardless of their country of origin and year of isolation, include Tn*6171* in precisely the same chromosomal location as in D36, as do the closely related KL13 subset of the ST328 Iranian isolates (Table 1). However, the remaining Iranian isolates (KL18 subset) do not carry Tn*6171* due to a large chromosomal deletion of the region where Tn*6171* would be located. The cause of this deletion was not explored here. Hence, Tn*6171* has entered this clade early and would have been present in the progenitor.

### Tn*6552*, a related transposon in ATCC 17978

Our analysis showed that the *fbs* siderophore genes in Tn*6171* are closely related (>99 % DNA identity) to those that were found in early studies to be present only in *A. baumannii* ATCC 17978 [8–10, 13, 14]. Though those studies did not detect a transposon in ATCC 17978, we re-examined the genetic context of the fimsbactin production cluster in ATCC 17978 and found that it is within a Tn*7-*family transposon that is quite closely related to Tn*6171* (Fig. 1a). Because there are significant differences (see below), including that it lacks two genes and the *tnsD* gene is interrupted by a copy of ISAba12 flanked by a 9 bp TSD, this transposon was named Tn*6552*.

Tn*6552* is 45 140 bp long, located at bases 981 919–1 027 058 in the corrected ATCC 17978 chromosomal sequence (GenBank accession no. CP012004; locus_ids ACX60_04645 to ACX60_04815) and bases 2 989 666–2 944 540 in the original (CP000521; locus ids A1S_2550 to A1S_2584), and is surrounded by a 5 bp TSD. It is bounded by the same 29 bp IRs as those found in Tn*6171*, and the same additional transposase binding sites as in Tn*6171* were also detected near the Tn*6552* boundaries. However, Tn*6552* is not found near *glmS*. Instead, it is located between ACX60_04635 and ACX60_04820, encoding a phosphoribosylformylglycinamidine synthase and a hypothetical protein, respectively, and this is likely due to the inactivation of the *tnsD* gene by the ISAba12. The *tns* genes associated with this transposon were noted previously [30].

Whilst the large segment of Tn*6552* that includes the iron-acquisition genes is 99.7 % identical at the DNA level to the corresponding region of Tn*6171*, elsewhere there are three recombinant regions with identities ranging from 88 to 97 % (Fig. 1a), presumably acquired from more distantly related transposons. ATCC 17978 belongs to ST437 and Tn*6552* was found only in the draft genomes of several further ST437 isolates from China (Table 2).

### Tn*6171* and closely related transposons in complete genomes

**Fig. 3.**
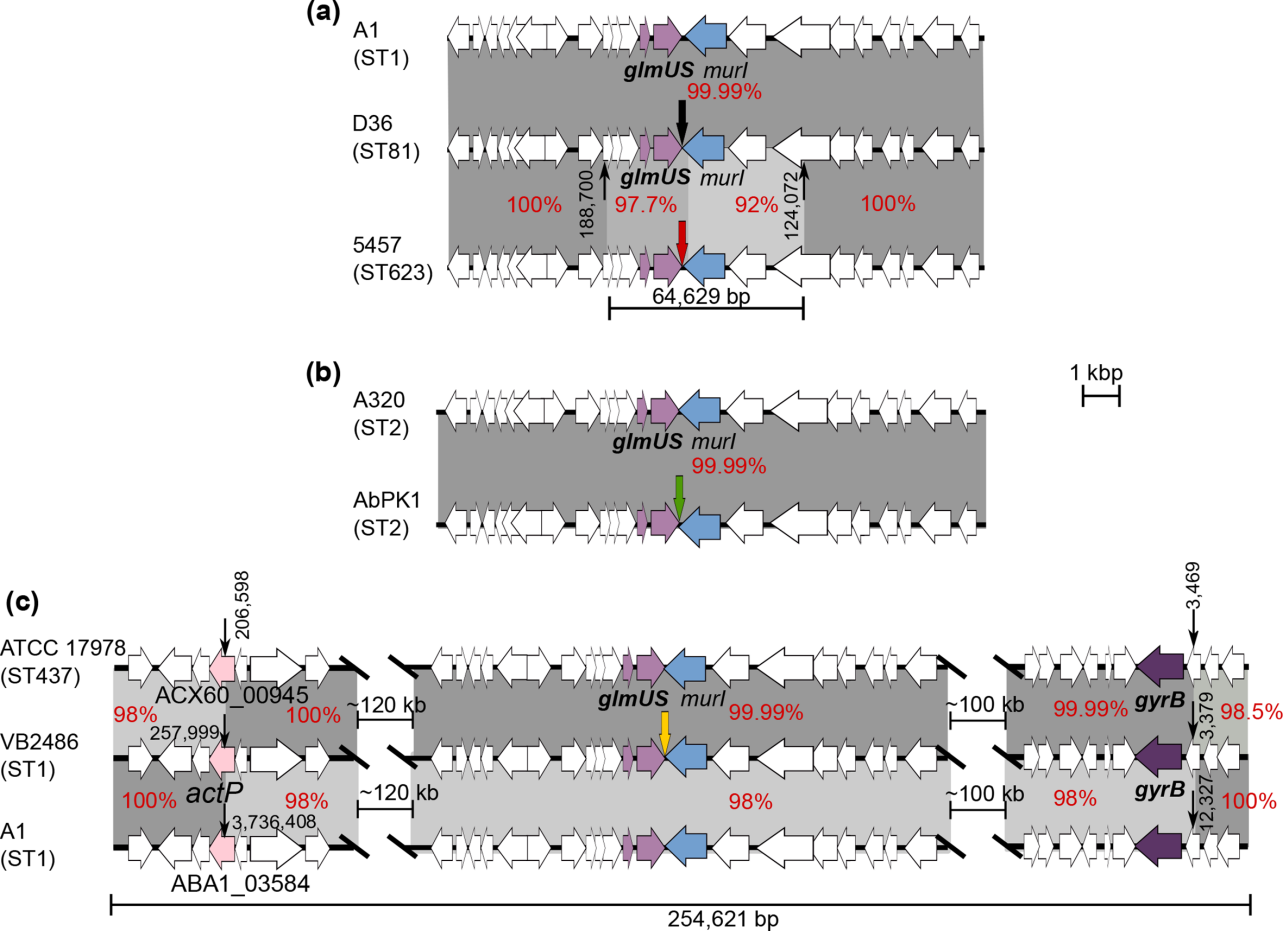
Schematic representation of the regions surrounding the *glmS* gene in (**a**) D36 (GenBank accession no. CP012952) compared to A1 (GenBank accession no. CP010781) and 5457, (**b**) A320 (GenBank accession no. CP032055) compared to AbPK1 (GenBank accession no. CP024576), and (**c**) VB2486 (GenBank accession no. CP050403) compared to ATCC 17978 (GenBank accession no. CP012004) and A1. Horizontal arrows indicate the extent and orientation of genes, with *glmSU* shown using a lavender colour and *murI* in blue. Grey shading indicates regions with significant DNA identities with their percentage identities shown in red. In (a) and (c), the extents of recombination patches in bp are shown underneath the figure. Thick coloured vertical arrows indicate the position of Tn*6171* or variants, with each colour indicating a different variant. Numbers next to the thin black vertical arrows indicate positions of recombination crossovers in the relevant chromosome.

To examine the distribution of Tn*6171*, the GenBank non-redundant database was queried using the sequence of Tn*6171* (last search September 2020) to find additional cases where the transposon is present. Tn*6171* was not present in any other complete genome, but a copy interrupted by an ISAba1 in the segment between ISAba12 and ∆IS*1236-var* was detected (Fig. 1b). This Tn*6171* variant (Tn*6171*::ISAba1) was found in *A. baumannii* AbPK1, as noted elsewhere [31]. AbPK1, recovered in Pakistan in 2012, represents a highly virulent GC2/ST2 strain that caused a serious outbreak of pneumonia in sheep [31]. Tn*6171*::ISAba1 is 51 200 bp long and is also downstream of *glmS* surrounded by an AAGGC duplication (Table 1). It also differs from Tn*6171* by the presence of 15 copies of an 8 bp unit found only three times in Tn*6171* in D36. The location of this repeat segment is indicated by a vertical red arrow in (Fig. 1b, c). Tn*6171*:ISAba1 appears to have entered this strain by transposition as the surrounding sequence is identical to that of A320, an early GC2 reference isolate [26], and most GC2 isolates (Fig. 3b).

**Fig. 4.**
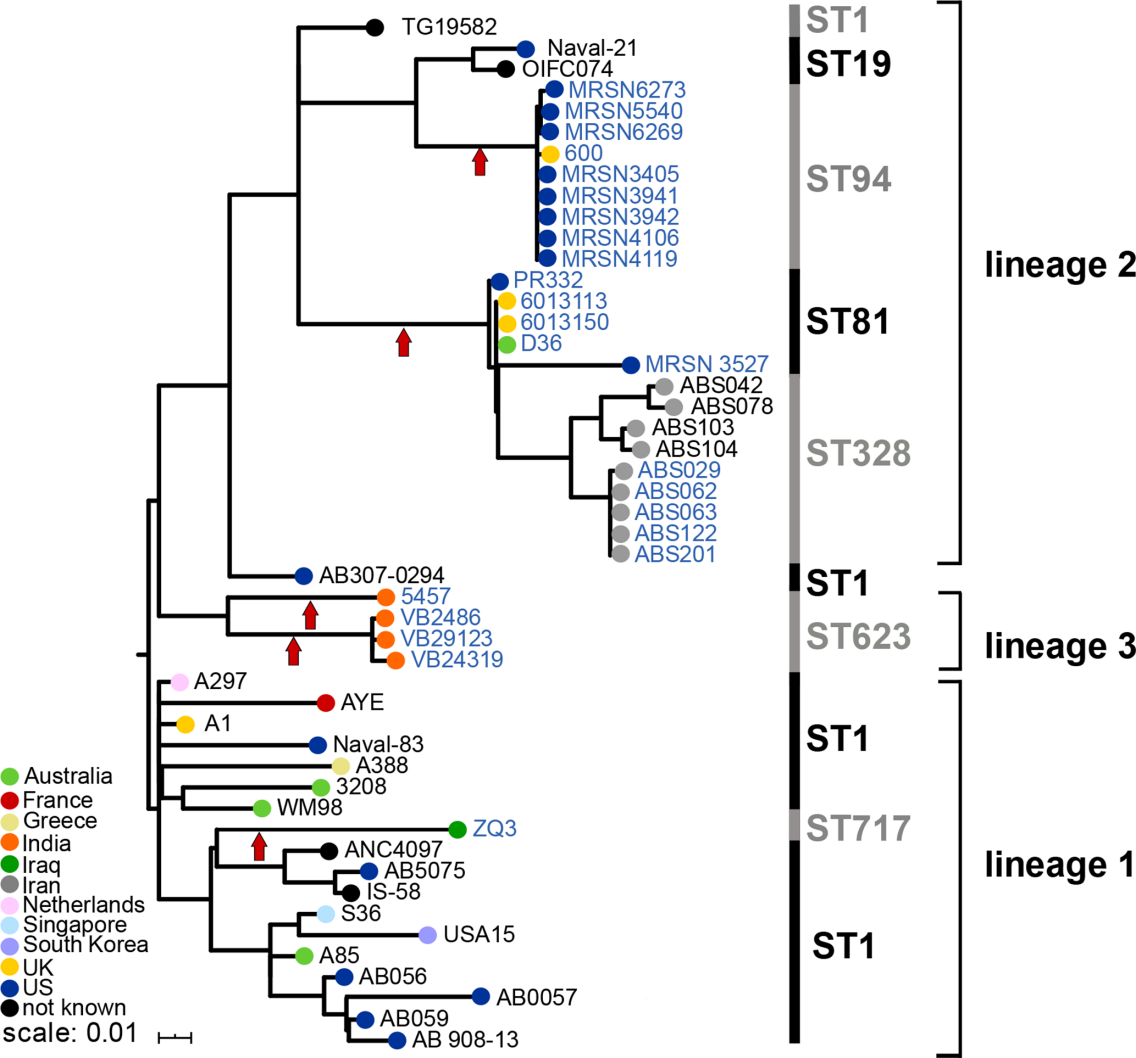
Whole-genome recombination-free phylogenetic tree of GC1s. Coloured nodes indicate the country of origin (see the key in the bottom left corner). Vertical red arrows indicate the acquisition of Tn*6171* or a related transposon. Institut Pasteur STs for each strain or strain set are indicated next to the black/grey vertical lines on the right of the figure. Lineages are also shown using square brackets. Lineage 2 strains with MRSN codes were isolated in the USA from soldiers recently returned from Afghanistan. The scale bar shown indicates the number of substitutions per site.

Two variants of Tn*6171* were identified, and were named Tn*6171*-v1 and Tn*6171-*v2 (Fig. 1b, c). Tn*6171*-v1, found in the chromosome of *A. baumannii* isolate 5457 recovered in India (GenBank accession no. CP045541), is 49 124 bp long and is also located downstream of *glmS* surrounded by the 5 bp AAGGC duplication (Table 1). It appears to be a deletion variant of Tn*6171* resulting from an ISAba12-mediated deletion that removed 791 bp of adjacent DNA sequence but also has 10 copies of the 8 bp repeat sequence. Isolate 5457 is ST623, a single locus variant of ST1; thus, it also belongs to GC1. However, in a recombination-free SNP-based phylogeny of selected GC1 genomes (Fig. 4), it is not part of lineage 2. Indeed, it appears to represent a third GC1 lineage. Moreover, when the DNA sequence surrounding Tn*6171*-v1 was compared to that in the early reference ST1 (GC1) isolate A1 [25] or surrounding Tn*6171* in D36, it was found to differ significantly from the standard GC1 sequence. The diverged sequence is 97.7 % identical on the left and extends for ~10 kbp. On the right, the level of divergence is much greater, potentially indicating an origin in a non-*baumannii Acinetobacter* isolate, and extends for ~6 kbp (Fig. 3a). Hence, a 16 kbp chromosomal segment has been replaced by the same segment, possibly containing the transposon (64.6 kbp total including the transposon), from a more distantly related isolate, and the transposon may have entered via a homologous recombination event rather than by transposition. The replacement sequence is referred to as the 623 region hereafter.

The second variant form, Tn*6171-*v2, is in VB2486, a GC1/ST1 strain recovered in 2019 in India. It is 52 602 bp and again located downstream of *glmS* but surrounded by a distinct ACAGC duplication (Table 1), indicative of an independent transposition event. It carries 18 copies of the 8 bp repeat and differs from Tn*6171* at the right end where segments of ISAba16 (2065/2552 bp) and an ISAba17-*var*∆ (447/2594 bp) are followed by a segment of about 1.5 kbp with 97 % DNA identity to the corresponding region in Tn*6171* (Fig. 1c). Notably, this segment was found to be almost identical, with only two base changes, to the corresponding part of Tn*6552*. However, the sequences of the chromosomal genes flanking Tn*6171-*v2 (*glmS* and HBN_00435) again differed significantly from the standard GC1 sequence. In fact, they were identical to the *glmS* region in ATCC 17978, which is not interrupted (Fig. 3c). This recombination patch is large, extending for 189 kbp to the left and 124 kbp to the right, after which the sequence returns to that most commonly present in GC1 isolates. Thus, Tn*6171-*v2 might have been acquired from a strain closely related to ATCC 17978 that carried Tn*6171-*v2 in *glmS*. As shown in Fig. 4, VB2486 is, like 5457, a member of the new lineage 3 of GC1, but it has acquired the transposon via a distinct event. Two further draft genomes of isolates carrying Tn*6171*-v2 (WGS accession numbers RHLV and RBVT) are from the same study as the complete genome of VB2480 and are also in the 437 context.

### Distribution of Tn*6171* in draft genomes

**Fig. 5.**
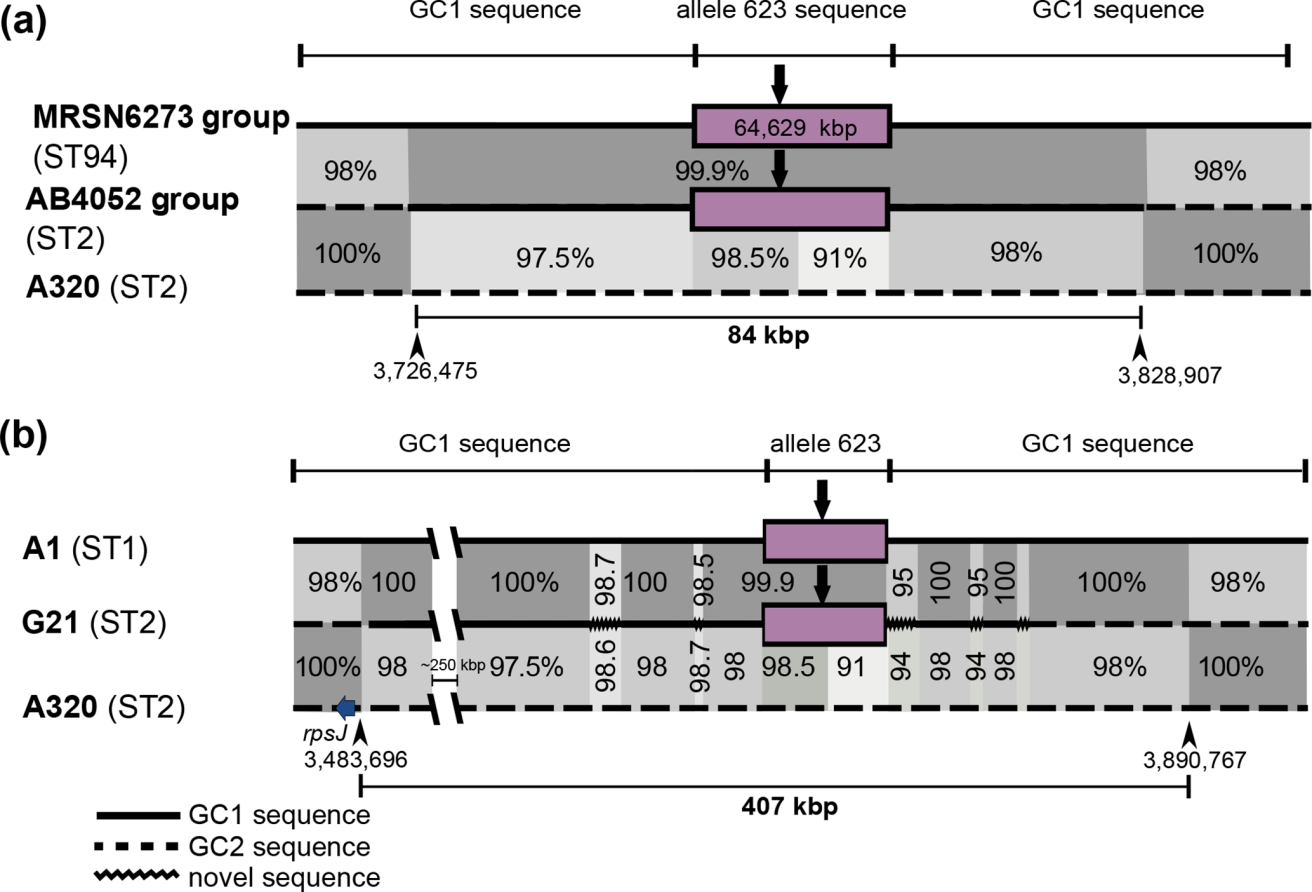
Schematic representation of the region surrounding the *glmS* gene in (**a**) AB4052 (ST2) (NCBI draft genome accession no. LRED) compared to MRSN6273 (ST94) (NCBI draft genome accession no. LNCY) and A320 (ST2) (GenBank accession no. CP032055), and (**b**) G21 (ST2) (NCBI draft genome accession no. UCOP) compared to A1 (ST1) (GenBank accession no. CP010781) and A320 (ST2). Black central lines indicate the chromosome, with continuous lines showing GC1 sequence, dashed lines GC2 sequences and zigzagged lines representing novel sequences. Central boxes coloured lavender represent the 623 patch, with a thick vertical arrow representing the position of Tn*6171* or variant. Shades of grey indicate regions with significant DNA identities and black numbers the DNA identities between the various chromosomal segments. Numbers next to the black arrowheads indicate chromosomal positions. Figure is not drawn to scale.

A database containing 3575 *A. baumannii* draft genomes, assembled previously [24], was also searched to find further examples of Tn*6171* or its variant forms. Tn*6171* with an additional copy of the 8 bp repeat was found in the genome of a further GC1/ST1 isolate, three GC2/ST2 isolates, as well as nine GC1/ST94 isolates and a single ST10 isolate (Table 1). The sequences surrounding the transposon in the ST1 isolate, R1B (not shown in Fig. 4), and the ST10 isolate, ARLG1323, were as found in most other isolates with the same ST. In contrast, in the ST2 (GC2) and ST94 (GC1 lineage 2) isolates, the transposon is surrounded by the 623 sequence found in isolate 5457 carrying Tn*6171*-v1, described above. The ST94 isolates, which were recovered in the USA from soldiers who had recently returned from Afghanistan [32, 33], carried the same 623 patch seen in 5457, which was isolated in India. However, the phylogeny places the ST94 isolates in lineage 2 rather than lineage 3 (Fig. 4). The ST2 isolates (ABBL040 and ABBL062) also carried the 623 patch flanked on both sides by GC1-derived sequence before returning to the expected GC2 sequence (Fig. 5a), suggesting that the transposon was acquired from a 5457-like isolate or an ST94 isolate.

### Distribution of Tn*6171*-v1 in draft genomes

The draft genome database also included further examples of Tn*6171*-v1 (Table 1). Tn*6171*-v1 was found in both GC1 and GC2 isolates. It is in a single GC1/ST1 isolate (ZQ3) surrounded by GC1 sequence and ZQ3 belongs to lineage 1 (Fig. 4), making it representative of a further independent acquisition of Tn*6171* by a GC1 isolate.

Two independent cases of acquisition of Tn*6171*-v1 were found in the GC2 clonal complex. In two GC2/ST2 isolates from Saudi Arabia, the transposon was found to be surrounded by GC2 sequence with a distinct 5 bp TSD of ACTAG (Table 1). A third GC2 isolate recovered in Australia from a returned soldier [34] also contains Tn*6171*-v1, but in the 632 context. Pairwise comparisons of the surrounding sequences revealed a complex relationship between the sequences in this isolate and in others containing the transposon in the 623 context, indicative of a series of recombination events involving different lengths of the surrounding sequence (Fig. 5b).

## Discussion

The two related transposons (and variant forms) described here exhibit features that make them members of a broader Tn*7* family. Specifically, the *tns* gene products are related to TnsABCDE that facilitate Tn*7* movement, and the end structures are related to those of Tn7. Tn*7* is one of the most studied transposons and is known to exhibit target specificity [16–18]. It targets a site downstream of the chromosomal *glmS* gene with a high frequency [17, 18] and, consistent with this, Tn*6171* and its variants were found near the 3'-end of the *A. baumannii* chromosomal *glmS* gene. However, Tn*6552* was not found in this location, likely due to the inactivation of the *tnsD* targeting determinant.

To the best of our knowledge, Tn*6171* and Tn*6552* are the first Tn*7*-family transposons that encode a siderophore production and uptake system to be reported, adding genes encoding iron-sequestration functions to the genetic material that is carried by members of this transposon family. The presence of the fimsbactin gene cluster in a Tn*7* family transposon means that this additional iron-acquisition system can move into new strains without the cost of interrupting important genes. However, the benefit of acquiring this siderophore system remains to be established experimentally.

This study confirms that the Tn*6171*-associated genes for fimsbactin siderophore synthesis and uptake are relatively rare. They were found only in *A. baumannii* genomes, and were present in a very small fraction of the hundreds of complete and thousands of draft *A. baumannii* genomes currently available in the NCBI GenBank nucleotide or WGS databases. Nonetheless, there was good evidence for repeated acquisition of Tn*6171* or one of its variants by isolates in both the GC1 and GC2 clonal complexes, which include the majority of multiply, extensively and pan-resistant isolates recovered in the clinical situation.

Granted that Tn*7* is known to transpose very efficiently into its preferred target site, the most surprising aspect of the analysis was the finding that the Tn*6171* had been acquired both by targeted transposition and by replacement of the target region, presumably already carrying the transposon, with the corresponding sequence from another source. *A. baumannii* is known to exhibit high levels of competence [35–37] and extensive replacement of specific chromosomal segments in members of a specific clonal complex is increasingly reported. Multiple instances of introduction of an IS upstream of the *ampC* gene leading to cephalosporin resistance have been recorded [38–40]. Replacement of the larger regions responsible for the production of surface polysaccharides is particularly common [4, 41, 42]. Also, a few cases of introduction of large resistance islands together with flanking sequence from their source have also been reported recently [40, 43]. A further case was noticed in the course of this study as the ST94 GC1 isolates all carry a resistance island in *comM* that is of the AbGRI1 type characteristically found in of CG2 isolates. Examination of the surrounding sequence revealed that the island had indeed been imported from a GC2 isolate.

## Supplementary Data

Supplementary material 1
