# Supplementary material for: Dissemination of novel Tn7 family transposons carrying genes for synthesis and uptake of fimsbactin siderophores among Acinetobacter baumannii isolates
Source: Microb Genom. 2021 Mar 22;7(3):mgen000548. doi: 10.1099/mgen.0.000548 (PMC8190619; doi:10.1099/mgen.0.000548)
Supplement: Supplementary material 1 [file mgen-7-0548-s001.pdf]

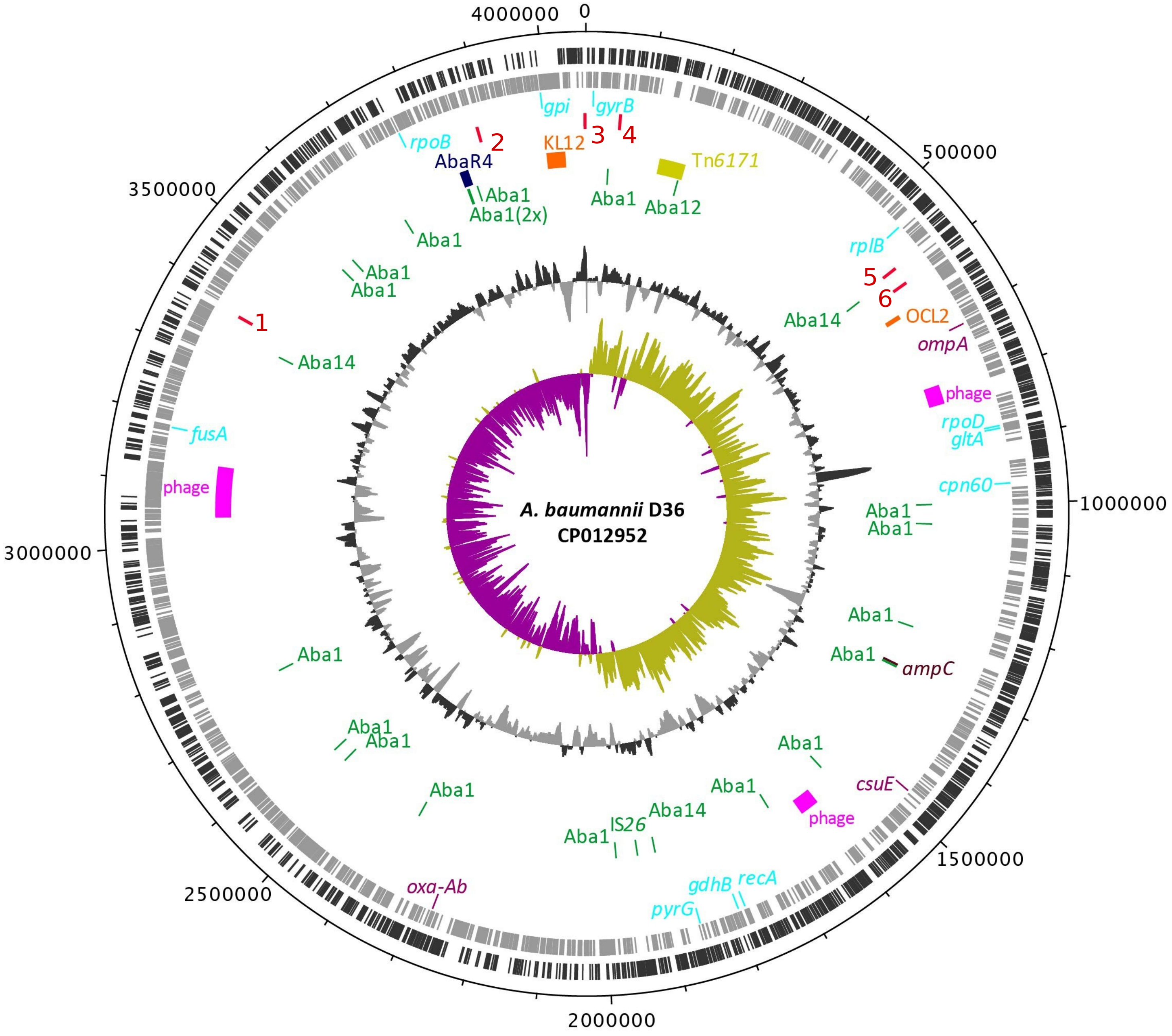

Fig. S1. Circular map of the chromosome of *A. baumannii* D36. The outermost ring (ring 1) represents the chromosome with small bars each representing 100 kbp length. Ring 2 and 3 (shown black and grey) represent open reading frames on the positive and negative strains, respectively. Ring 4 (shown grey and black) and 5 (represented mauve and lime) represent GC content and GC skew, respectively. The chromosomal locations of genes used in MLST schemed (Institut Pasteur and Oxford schemes) are shown using small cyan bars with the gene names indicated. All insertion sequences found in the chromosome of D36 are also shown using small green bars with the IS names indicated next to each bar. Prophage genomes, KL and O loci are also shown

using pink and orange boxes with their names appear next to them. Small red bars numbered 1-6 also represent the chromosomal locations of ribosomal RNA (rRNA) genes found in the chromosome of D36. D36 has an inversion between rRNA genes 3 and 4.

Table S1. Proteins encoded by Tn*6171*[a].

Coordinates [b]	locus id (AN415)	Product	Size (aa) [c]	Pfam	Function
182801-183610c	00167	TnsA	270	08721-2	Transposition protein
180727-182730c	00166	TnsB	668	665	Transposition protein
179081-180730c	00165	TnsC	550	11426	Transposition protein
177536-179065c	00164	TnsD	510	15978	Transposition protein
175960-177546c	00163	TnsE	529	18623	Transposition protein
175497-175724	00162	Tnp1236	76	13276	Putative transposase
175121-175285c	00161	Orf	55	NA	Unknown
174805-174933c	00160	Orf	43	NA	Unknown
174591-174773c	00159	Orf	61	NA	Unknown
173894-174232c	00158	Orf	113	NA	Unknown
173396-173620c	00157	Orf	75	NA	Unknown
172456-173325c	00156	TnpAba12	290	13737	Transposition protein of ISAba12
171766-172041c	00155	Orf	92	NA	Unknown
170540-171688c	00154	RoxA	383	8007	Involved in ribosomal translation, structure and biogenesis
169266-170531c	00153	HlyD	422	529	membrane-fusion protein of T1SS
167145-169262c	00152	SunT	706	3412	Putative ABC-type bacteriocin/antibiotic exporter
165586-166941	00151	FbsQ	452	1554	Multidrug resistance Efflux pump
164443-165567	00150	FbsP	375	4954	Putative siderophore-interacting protein; ferric reductase
163243-164391	00149	FbsO	383	7690	Putative MFS efflux pump
160916-163123	00148	FbsN	736	593	Ferrichrome-iron receptor
159932-160693c	00147	FbsM	254	975	Putative iron acquisition Pr; similar to Yersiniabactin synthesis enzyme
159048-159917c	00146	FbsL	290	1648	Phosphopantetheinyl transferase
158534-158887	00145	Orf	118	NA	Unknown
158380-158559	00144	Orf	60	NA	Unknown
157388-158014	00143	FbsK	209	13523	Siderophore synthetase small component; acetyltransferase
156146-157306	00142	FbsJ	387	2784	Ornithine/Arginine decarboxylase
154796-156136	00141	FbsI	447	13434	Siderophore biosynthesis protein, monooxygenase
153163-154782	00140	FbsH	540	501	2,3-dihydroxybenzoate-AMP ligase
149936-153151	00139	FbsG	1072	668	Siderophore biosynthesis non-ribosomal peptide synthetase module
146687-149923	00138	FbsF	1079	501	Siderophore biosynthesis non-ribosomal peptide synthetase module - AMP-binding enzyme
144179-146683	00137	FbsE	835	550	Peptide synthetase similar to enterobactin
143374-144165	00136	FbsD	264	13561	2,3-dihydro-2,3-dihydroxybenzoate

					dehydrogenase
142708-143337	00135	FbsC	210	857	Isochorismatase (hydrolase) of siderophore biosynthesis
141441-142616	00134	FbsB	392	425	Isochorismate synthase of siderophore biosynthesis
140138-141064c	00133	FbsA	309	12833	Transcriptional regulator AraC-type
137954-139753c	00132	KAP	600	7693	Putative NTPAse
137178-137939c	00131	Orf	254	NA	Unknown
135863-137185c	00130	Orf	441	NA	Unknown
135123-135866c	00129	TatD	248	1026	Putative DNAse
133985-134698c	00128	Orf	238	NA	Unknown

[a] located at bases 133828-183749 of GenBank accession no. CP012952.

[b] based on the D36 genome (GenBank accession no. CP012952).

[c] protein size (amino acid).
